# Aromatase Inhibitors and Dementia Risk: Putting Safety Into Perspective

**DOI:** 10.1002/prp2.70104

**Published:** 2025-05-02

**Authors:** Mariam Rizk, Kefah Mokbel

**Affiliations:** ^1^ The London Breast Institute Princess Grace Hospital London UK

Dear Editor,

We read with interest the recent study investigating a potential association between third‐generation aromatase inhibitors (AIs) and dementia using VigiBase data [1]. While the authors present a compelling case for further exploration of this link, several key methodological limitations were either inadequately discussed or overlooked.

First, pharmacovigilance databases like VigiBase are inherently prone to reporting bias. The underreporting of adverse drug reactions (ADRs) is well recognized, particularly for chronic, insidious conditions such as dementia. Conversely, heightened awareness of cognitive issues in breast cancer survivors undergoing AI therapy may contribute to selective overreporting, artificially inflating the observed signal. Without robust denominator data, disproportionality analyses alone cannot establish causality or even true incidence rates.

Second, confounding variables were not sufficiently controlled. Age, baseline cognitive function, comorbidities (e.g., cerebrovascular disease, diabetes, depression), and concurrent medications (e.g., antihypertensives, statins, or other endocrine therapies) are critical contributors to dementia risk. The authors acknowledge this limitation but do not provide any sensitivity analyses or adjustments to mitigate these confounding effects.

Third, indication bias remains a major concern. AIs are prescribed exclusively to postmenopausal women, a group inherently at increased risk for dementia due to estrogen decline and aging itself. The study compares AI users to all other drug users in the database rather than an appropriate control group of postmenopausal women not receiving AIs. Without such a comparator, the reported RORs may reflect background dementia risk rather than a drug effect.

Moreover, the lack of longitudinal follow‐up and missing data on treatment duration further weaken the conclusions. The study reports that in 86%–96% of cases, treatment duration was unknown, and in most reports, no information was available on whether cognitive symptoms improved after discontinuation. This is critical, as transient cognitive impairment related to cancer treatment (“chemo brain”) could be misclassified as early dementia.

Finally, the absence of validation through large‐scale epidemiological studies is a key gap. While pharmacovigilance signals are useful for hypothesis generation, they must be corroborated by prospective cohort studies or randomized trials before clinical implications can be drawn.

Conversely, other studies have found no significant impact of AIs on cognitive function. Ilhan et al. reported that prolonged adjuvant treatment with AIs does not affect cognitive function in hormone receptor‐positive breast cancer patients [2]. Additionally, some research indicates that AIs might have protective effects against neurodegenerative diseases. A study in JAMA Network Open found that tamoxifen and AIs were associated with significantly reduced relative risks for all neurodegenerative diseases, including Alzheimer's disease [3]. These findings directly challenge the conclusions drawn in the current study and highlight the need for caution in interpreting pharmacovigilance signals without robust epidemiological validation.

Beyond methodological concerns, the clinical impact of such findings cannot be ignored. Breast cancer patients often face difficult treatment decisions, and undue alarm over unproven risks may deter women from taking life‐saving endocrine therapy. Given the well‐established benefits of AIs in reducing recurrence and improving survival, it is crucial that such studies provide a balanced interpretation of findings, emphasizing limitations to avoid unwarranted fear and nonadherence.

In conclusion, while this study raises an important question regarding AI use and dementia risk, its methodological shortcomings significantly limit the strength of its conclusions. Future research should integrate prospective cognitive assessments, better‐matched control groups, and confounder‐adjusted analyses to clarify this potential relationship. Until then, patients and clinicians should interpret these findings with caution, ensuring that concerns about unproven risks do not compromise adherence to proven, life‐extending treatments.

## Author Contributions

K.M. conceptualization. K.M. writing – original draft preparation. M.R. and K.M. writing – review and editing. All authors have read and agreed to the published version of the manuscript.

## Conflicts of Interest

K.M. received research grant provided by Breast Cancer Charity, consulting fees from QMedical and Sebbin, and honoraria for offering academic and clinical advice to Merit Medical and QMedical corporations. He is a fractional shareholder of Datar Genetics stock. Furthermore, he owns shares in HCA Healthcare UK and OncoBotanica. The other author has no conflicts of interest to declare.

## Data Availability

The data presented in this study are publicly available and can be used without restrictions.
